# Microencapsulation, Chemical Characterization, and Antimicrobial Activity of Mexican (*Lippia graveolens* H.B.K.) and European (*Origanum vulgare* L.) Oregano Essential Oils

**DOI:** 10.1155/2014/641814

**Published:** 2014-08-06

**Authors:** Elvia Hernández-Hernández, Carlos Regalado-González, Pedro Vázquez-Landaverde, Isabel Guerrero-Legarreta, Blanca E. García-Almendárez

**Affiliations:** ^1^DIPA, PROPAC, Facultad de Química, Universidad Autónoma de Querétaro. C. U., Cerro de las Campanas s/n, Col. Las Campanas, 76010 Querétaro, QRO., Mexico; ^2^Centro de Investigación en Ciencia Aplicada y Tecnología Avanzada, Instituto Politécnico Nacional Unidad Querétaro, Cerro Blanco 141, Colinas del Cimatario, 76090 Querétaro, QRO., Mexico; ^3^Universidad Autónoma Metropolitana-Iztapalapa, San Rafael Atlixco No. 186, Col. Vicentina, Iztapalapa, 09340 México, DF, Mexico

## Abstract

The effect of solvent polarity (methanol and pentane) on the chemical composition of hydrodistilled essential oils (EO's) of *Lippia graveolens* H.B.K. (MXO) and *Origanum vulgare* L. (EUO) was studied by GC-MS. Composition of modified starch microencapsulated EO's was conducted by headspace-solid-phase microextraction (HS-SPME). The antimicrobial activity of free and microencapsulated EO's was evaluated. They were tested against *Salmonella* sp., *Brochothrix thermosphacta*, *Pseudomonas fragi, Lactobacillus plantarum*, and *Micrococcus luteus*. Thymol and carvacrol were among the main components of EO's and their free and microencapsulated inhibitory activity was tested against *M. luteus*, showing an additive combined effect. Chemical composition of EO's varied according to the solvent used for GC analysis and to volatile fraction as evaluated by HS-SPME. Thymol (both solvents) was the main component in essential oil of MXO, while carvacrol was the main component of the volatile fraction. EUO showed *α*-pinene (methanol) and *γ*-terpinene (pentane) as major constituents, the latter being the main component of the volatile fraction. EO's showed good stability after 3 months storage at 4°C, where antimicrobial activity of microencapsulated EO's remained the same, while free EO's decreased 41% (MXO) and 67% (EUO) from initial activity. Microencapsulation retains most antimicrobial activity and improves stability of EO's from oregano.

## 1. Introduction

Originally added to improve food taste and/or color, aromatic plants can also enhance shelf life of foods because of their antimicrobials content and antioxidant properties [1]. Recently, consumers demand for natural food ingredients has increased because of their safety and availability [2]. Essential oils (EO's) are a mixture of volatile compounds obtained from plant materials (flowers, buds, seeds, leaves, and among others) [3] that have shown wide antimicrobial activity spectra and may act as natural food preservatives when added to fresh food products [4]. Antimicrobial properties have been associated to their composition, structure, and functional groups [5, 6]. The EO's of European oregano (*Origanum vulgare* L., EUO) and Mexican oregano (*Lippia graveolens* H.B.K., MXO) are active against bacteria, yeasts, and molds, in which thymol and carvacrol are mainly responsible for these properties [4, 7, 8]. These compounds disrupt the cell membrane, causing an increased permeability/disintegration. Damage is reflected on the dissipation of the two components of proton motive force, pH gradient, and electrical potential [9]. In addition, thymol can up- or downregulate genes involved in outer membrane protein synthesis [10].

Nevertheless, antimicrobial activity could be influenced by the composition and quantity of EO's bioactive molecules as affected by geographical origin, variety, growth conditions, seasonal variations, vegetative cycle, environmental and soil factors, storage time, and leaves drying method [11, 12]. There are more than 40 different classes of herbs known as oregano [13].

Headspace- (HS-) solid-phase microextraction (SPME) is another technique to study the composition of the volatile fraction of EO's. HS-SPME is a solvent-free technique used to sample the gaseous or volatile phase in equilibrium with a solid matrix to characterize its composition [14]. EO's may be microencapsulated to protect them from light, air, and humidity, despite partial reduction in biological activity due to volatilization, oxidation, or interactions with encapsulating material. Wall material, process type, and temperature are factors that significantly influence the antimicrobial activity of microencapsulated EO's [15, 16], which can be used for the controlled release of biologically active substances at a specific action site.

The objective of this work was to characterize the chemical composition of free and microencapsulated EO's from* Origanum vulgare *L. and* Lippia graveolens *H.B.K. leaves and to determine their antimicrobial activity against spoilage and pathogenic bacteria commonly found in fresh foods, such as* Brochothrix thermosphacta, Lactobacillus plantarum, Pseudomonas fragi*,* Salmonella* sp., and* Micrococcus luteus *NCIB 8166. We also aimed to determine the combined antimicrobial effect of two of their main components, thymol and carvacrol.

## 2. Materials and Methods

### 2.1. Chemicals

Methanol, sodium chloride, and potassium chloride were purchased from J.T. Baker (Xalostoc, Estado de México, México), while pentane was purchased from Eastman (Kingsport, TN, USA). Anhydrous sodium sulfate, sodium bicarbonate, thymol, carvacrol, Tween 20, and Tween 80 were obtained from Sigma-Aldrich (St. Louis, MO, USA), whereas anhydrous calcium chloride was supplied from Merck (Darmstadt, Germany).

### 2.2. Plant Material and Extraction of EO's

EUO leaves were collected from Santiago Mamalhuazuca (Estado de México, México) 24 h before utilization and oven-dried at 35°C for 24 h. MXO leaves and flowers were harvested and sun-dried in Toliman (Querétaro, México). Dry material was stored in black polyethylene bags at 25°C until use.

EO's were obtained by hydrodistillation of 270 g of dry MXO and 800 g of dry EUO, with 5 L of distilled water for 3 h, using a Clevenger-type apparatus (Cristalab, DF, México) [17]. The oily layer (11.6 and 5.2 mL for MXO and EUO, resp.) on top of the aqueous distillate was removed and dried with anhydrous sodium sulfate. The EO's were stored in sealed vials protected from light at 4°C until further analysis.

### 2.3. GC/MS Analysis of Oregano EO's

GC/MS analyses were conducted using a GC (model 7890A, Agilent Technologies, Santa Clara, CA, USA) equipped with an MPS2XL autosampler (Gerstel GmbH, Germany) and coupled to a mass spectrometer detector (model 5975, Agilent Technologies). A HP-5 capillary column 60 m long × 0.25 mm internal diameter and 0.25 *μ*m film thickness (Agilent Technologies) was used. Ultrapure Helium (Infra, Querétaro, México) was used as carrier gas at a flow rate of 1.0 mL/min and injector temperature of 300°C. EO's were filtered through a 0.22 *μ*m membrane (Millipore, MA, USA) and diluted (1 : 10, v/v) with methanol or pentane. Two *μ*L was injected under split mode (ratio 1 : 50). Oven temperature program was 40°C for 10 min, rising at 5°C/min to 230°C, followed by rising at 20°C/min to 300°C, and held for 10 min. Ionization potential for mass spectrometry was kept at 70 eV, while electronic ionization source and quadrupole temperatures were 230°C and 150°C, respectively. Total ion monitoring was done using a scan mass range of 33–900 m/z. Quantification was calculated as the ratio of peak area of each compound to total chromatographic area. Compounds identification was conducted by comparing the obtained mass spectra (MS) with those of the NIST 2010 library.  Table 1 shows components with at least 80% similarity with the library of spectra. Confirmative identification was carried out by determining their Kovats retention indices (KIs), achieved by injecting a solution containing a series of n-alkanes (C5–C29) (Sigma Aldrich) at same chromatographic conditions as EO's. The obtained KIs were compared with those reported in the literature.

### 2.4. Microencapsulation of EO's, Thymol and Carvacrol

Modified starch (Ingredion, Bridgewater, NJ, USA) was used as wall material at 28.6% w/w and to emulsify 16% w/w oregano EO's, thymol or carvacrol. Modified starch was dissolved in deionized water at 50°C stirring overnight. Antimicrobials were slowly added to the starch dispersion, while being homogenized at 10,000 rpm using an Ultra Turrax (T25, Wilmington, NC, USA) at ambient temperature, and, after antimicrobials incorporation, homogenization continued for 6 min. Thymol was dissolved in 10% (v/v) Tween 80 solution. The emulsions were dried using a minispray-dryer (Büchi B-191, Switzerland) with inlet air temperature of 190°C and exit temperature of 100°–110°C. Particle size was analyzed using a Mastersizer (Malvern Instruments, Model 2000, Worcestershire, UK). The powder product was stored in a sealed container protected from light at 4°C until use.

### 2.5. Headspace Volatile Compounds Analysis

Volatile compounds of MXO and EUO EO's microcapsules were identified using headspace- (HS-) solid-phase microextraction (SPME) technique, coupled with gas chromatography and mass spectrometry [7]. HS-SPME technique is mainly based on sorption of volatiles accumulated in headspace onto polymeric fiber coating. For this study, 2 cm long bipolar carboxen-divinylbenzene-polydimethylsiloxane (DVB/CAR/PDMS) fiber (Supelco Technology, St. Louis, MO, USA) was used, which is among the most frequently used in aromatic plant analysis [14]. MXO or EUO microcapsules of EO's (100 mg) were mixed with 3 g of distilled water and placed on suitable vials; then, the headspace was contacted by the SPME device for 10 min at 40°C. Preliminary experiments determined that equilibrium was reached within this time. The fibers were then transferred to the injection port of the GC and were desorbed under splitless mode at 250°C. Chromatographic analysis (in triplicate) was performed under the same conditions used for GC/MS composition analysis of oregano EO's.

### 2.6. Scanning Electron Microscopy (SEM)

Morphology of EO's microcapsules was examined by scanning electron microscopy (ESEM Phillips, model XL30, Amsterdam, Netherlands). The microencapsulated samples were deposited onto specimen stubs, under low vacuum (119 Pa) and 50 *μ*A current.

### 2.7. Microorganisms Tested

All bacteria tested were obtained from the microbial collection of the Food Biotechnology Laboratory, DIPA, Universidad Autónoma de Querétaro, Mexico. Selected bacteria were those relevant in microbial contamination of fresh foods [18, 19]. Gram-positive:* Brochothrix thermosphacta* and* Lactobacillus plantarum*; Gram-negative:* Pseudomonas fragi* and* Salmonella* sp., while* Micrococcus luteus* NCIB 8166 was chosen as positive control because of its high sensitivity to the tested EO's. The strains were stored at −70°C in sterile skim milk and glycerol mixture. All bacteria were activated in nutrient broth (Bioxon, Estado de México, México) at 30°C for 24 h, except* Salmonella* sp. which was activated at 37°C.

### 2.8. Antimicrobial Activity of Free and Microencapsulated Thymol and Carvacrol against* Micrococcus luteus*

The antimicrobial effect of tested compounds was compared using the broth dilution method. Stock solutions of free and microencapsulated thymol and carvacrol (5% w/v) were prepared using 10% (v/v) Tween 80. The antimicrobial agent content in microcapsules was determined considering its total quantity in the modified starch emulsion and the recovered solid fraction after drying.

Tubes containing appropriate dilutions were inoculated to a final concentration of 10^5^ CFU/mL* Micrococcus luteus* suspension in nutritive broth. The tested concentration ranges were 0–250 *μ*g/mL for thymol and 0–500 *μ*g/mL for carvacrol, both free and microencapsulated. Tubes were then incubated for 2 h at 30°C, followed by population determination on nutritive agar by incubation at 30°C for 48 h, using the drop method (detection limit 1.7 log CFU/mL). All experiments were conducted in triplicate.

### 2.9. Combined Effect of Thymol and Carvacrol against* Micrococcus luteus*

Minimum inhibitory concentration (MIC) was determined for thymol and carvacrol. Concentrations varied from 0 to 1250 *μ*g/mL for thymol and 0–1000 *μ*g/mL for carvacrol, both were diluted with 10% (v/v) Tween 80. Tubes containing appropriate concentration proportions were inoculated to achieve 10^5^ CFU/mL* Micrococcus luteus* suspension in nutritive broth. After 8 h incubation at 30°C, population was determined by plating on nutritive agar and incubated at 30°C for 48 h. Assays were performed in triplicate. A checkerboard array of serial concentration proportions of the two antimicrobials was performed, and fractional inhibitory concentrations (FICs) were calculated. FICs were used to obtain the FIC_index_ defined as FIC_*I*_ = (MIC of antimicrobial A in combination/MIC of A alone) + (MIC of antimicrobial B in combination/MIC of B alone). If the FIC_*I*_ is <1, the interaction is synergistic; near to 1 indicates additive interaction, while >1 indicates antagonism [20].

### 2.10. Antimicrobial Activity of EO's

The disk diffusion method in agar was used to determine the antibacterial capacity of free and microencapsulated EO's [21]. Ten mL of soft nutrient agar (0.8% w/v, Bioxon) was mixed with 200 *μ*L each of 50% (v/v) Tween 20 and Tween 80 solutions to favor EO's diffusion; they may also facilitate microbial membrane and cell wall penetration [22]. Bacterial population was adjusted to 10^7^–10^8^ CFU/mL with peptone solution (0.1% w/v, Bioxon) (*B. thermosphacta, P. fragi, L. plantarum, *and* Salmonella* sp.) or quarter strength Ringer's solution (*M. luteus*). The suspension (0.1 mL) was added to the soft agar and then poured into plates containing solidified agar (1.5% w/v).

Antimicrobial activity of free or microencapsulated EUO and MXO essential oils diluted with 10% (v/v) Tween 80 was tested at two different concentrations (15% and 25%, w/v) acting on the tested spoilage and pathogenic bacteria.

Polyvinylidene fluoride (PVDF) membrane disks 25 mm in diameter (Darmstadt, Germany) were impregnated with 75 *μ*L of each dilution of filter-sterilized MXO or EUO EO's. One disk was gently placed on top of the soft agar layer and EO's were allowed to diffuse for 2 h at 4°C and then incubated at the appropriate temperature during 48 h, except* M. luteus* which was incubated for 120 h. The growth inhibition zone, which included the membrane diameter, was measured using vernier calipers. The effect of the surfactants was used as control.

### 2.11. Essential Oils Stability

Antimicrobial effect of free and microencapsulated EUO and MXO EO's at 25% concentration against* Micrococcus luteus* was determined using disk diffusion method in agar. These antimicrobials were stored in sealed vials protected from light at 4°C during 3 months, followed by antimicrobial activity evaluation.

### 2.12. Statistical Analysis

Concentration of compounds found for both types of oregano and antimicrobial activity were analyzed in triplicate and expressed as mean values. Data were analyzed using SPSS software version 20.0 (Chicago, IL, USA) and means comparison was performed using Tukey's test (*P* < 0.05).

## 3. Results and Discussion

### 3.1. Chemical Characterization of EO's

Oils obtained after hydrodistillation of both types of oregano showed a clear yellow color and strong characteristic smell. MXO essential oil showed a yield of 4.29% (w/w, dry basis) which is 6.6 times higher than that obtained from EUO essential oil (0.65% w/w, d.b.); these data agree with those previously reported [13]. A similar yield (4.1%) was reported from dried leaves, flowers, and small branches of* Lippia graveolens* harvested in Querétaro, Mexico [23]. In addition, a 0.45% (w/w) yield of EO from hydrodistilled dry leaves of* Origanum vulgare *was reported [24], which is lower than our results.

Table 1 shows the chemical composition of EUO and MXO essential oils diluted with both methanol and pentane, expressed as % peak area. Compounds were identified using the GC/MS library and by their KIs. The EO's chemical composition from both types of oregano was different, and, depending on the solvent used to dilute the same EO sample, different compounds were found. For instance, 56 compounds were identified for methanol diluted MXO, in which the main compound was thymol (66.3%), distantly followed by its biosynthetic precursor *γ*-terpinene (9.59%), *α*-pinene (4.85%), and *β*-thujene (2.08%). When MXO essential oil was diluted with pentane, 57 compounds were identified, thymol being the main compound (49.9%), followed by *γ*-terpinene (10.31%), *β*-thujene, *α*-terpinene, and *β*-myrcene (2.32% each one). Using any of the two solvents, MXO essential oil showed very low carvacrol peak area composition (0.1%).

Methanol dilution of EUO essential oil allowed identification of 51 compounds, among them *α*-pinene (15.56%), terpinen-4-ol (14.77%), thymol (9.3%), carvacrol (8.4%), and *α*-terpinene (6.95%). Main compounds found from pentane dilution of EUO were *γ*-terpinene (15.06%), terpinen-4-ol (12.4%), thymol (11.5%), *α*-terpinene (10.38%), and carvacrol (8.4%), from a total of 41 identified compounds. Thymol composition in MXO essential oil was at least 4.3 times higher than that found in EUO essential oil, which is in agreement with a previous report [2].

Compounds found in EO's of both types of oregano were classified in 5 groups (Table 1): monoterpene hydrocarbons, oxygenated monoterpenes, sesquiterpene hydrocarbons, oxygenated sesquiterpenes, and others (including hydrocarbons, aldehydes, ketones, ester, carboxylic acids, and aliphatic and cyclic alcohols). Both EO's comprised mainly monoterpenes with either hydrocarbonated or oxygenated structures: 48% and 51% for MXO essential oil, 60% and 66% for EUO essential oil using methanol and pentane dilutions, respectively. Thus, essential oil from EUO showed higher monoterpenes content than MXO irrespective of the solvent used.

Polarity index (PI), which is a relative measure of solvent degree of interaction with various test solutes, is 0.0 for pentane and 5.1 for methanol. Thus, the partition coefficient in the GC column of individual compounds depended on their solubility in each solvent [25]. This may explain the selective detection of individual compounds depending on the solvent used for the same EO, such as* cis*-sabinene-hydrate, eugenol and germacrene D in MXO essential oil and isocaryophyllene and* p*-cymene in EUO essential oil when diluted in pentane (Table 1). Other compounds were detected only for methanol dilution, such as *β*-ocimene and *β*-elemene in MXO essential oil and 3-carene and isothymol-methyl-ether in EUO essential oil. Composition was also affected by the solvent used because thymol in MXO essential oil was 66.3% peak area when diluted with methanol, whereas it was 49.9% when diluted in pentane. In relation to *α*-pinene, it was found in methanol diluted EUO essential oil at 15.56%, while pentane dilution yielded 10.38% peak area. Main compounds previously reported for MXO essential oil are thymol, carvacrol, and *p*-cymene [2], whereas, in this work, only thymol was within the most abundant compounds. EO's from plants of* Lippia graveolens* from different Mexican regions, harvested at different seasons and year, comprised mainly eucalyptol, thymol, and carvacrol for one lot, while a second lot showed that carvacrol, eucalyptol, and *β*-caryophyllene were the most abundant [7]. In contrast, our results show a very low carvacrol concentration for MXO essential oil irrespective of solvent used (Table 1).

Carvacrol, thymol, linalool, caryophyllene oxide, and germacrene-d-4-ol were the main compounds found in three different lots of EUO essential oil using diethyl ether (PI = 2.8) as solvent [26]. Carvacrol and *p*-cymene were the main compounds found when hexane was used as solvent (PI ≈ 0.0) [27]. In contrast, our results show that peak area of terpinen-4-ol was higher than that of thymol and carvacrol (Table 1). This difference in composition may be attributed to the region of origin [28], growth conditions, time of harvest, plants maturity, leaves drying method [7], and/or solvent used for GC/MS analysis.

### 3.2. Chemical Characterization of Microencapsulated EO's and Thymol and Carvacrol

EO's microencapsulation efficiency was 80.7% for MXO, whereas that for EUO was 73.7%, by weight.  Table 2 shows the chemical composition of microencapsulated EUO and MXO essential oils, showing that 30 volatile compounds were detected by both techniques HS-SPME and GC/MS of hydrodistilled essential oils. However, GC/MS analysis of hydrodistilled EO's cannot be directly compared with HS-SPME technique because the basic principles of the two approaches are different [7]. Thymol has shown affinity for modified starch which we used as wall material for spray drying, thereby reducing its release in water, and this may explain the reduced thymol peak area detected (Table 2) [29].

Solids recovery after drying was 77.9% (w/w) for thymol and 75.6% (w/w) for carvacrol, when using pure compounds, which led to microcapsules containing 22% thymol and 23.4% carvacrol, by weight.

### 3.3. Scanning Electron Microscopy (SEM)

**Figure 1:**
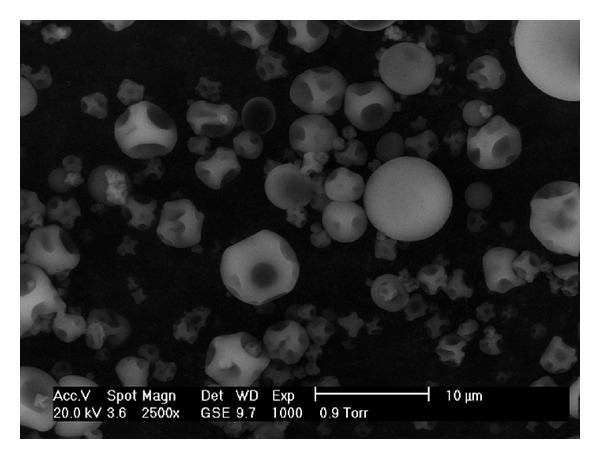
Micrograph of microencapsulated oregano EO, by spray drying. Magnification is 2,500x.

Microencapsulated particles of both EO's did not show cracks or pores. All particles showed a diversity of shapes from ovoid to spherical with surface dents (Figure 1). The size of microcapsules varied from 3 to 8 *μ*m. Size control of spray drying is an important factor because it influences appearance and dispersibility [30].

### 3.4. Antibacterial Activity of Free and Microencapsulated Thymol and Carvacrol against* Micrococcus luteus*

Both microencapsulated compounds were more effective in bacterial growth inhibition. This characteristic may be attributed to the increased solubility and bioavailability of these compounds [16]. The antimicrobial effect was initially observed after two hours of incubation, whereas higher concentrations of free and microencapsulated compounds produced increased antimicrobial effect (Table 3).

### 3.5. Combined Effect of Thymol and Carvacrol against* Micrococcus luteus*

Minimum inhibitory concentrations (MIC) were 1250 *μ*g/mL for thymol and 1000 *μ*g/mL for carvacrol. The FIC_*I*_ obtained was 1.03 ± 0.12 which indicates an additive effect between the two compounds (Table 4). This effect has also been suggested for a combination of thymol and carvacrol using* Staphylococcus aureus* and* Pseudomonas aeruginosa* as sensitive microorganisms [9].

### 3.6. Essential Oils Antibacterial Activity

EO's microencapsulation led to a powder showing a significantly lower smell than free EO's, while antimicrobial properties are significant (Table 5).* M. luteus* is the most sensitive of the bacteria tested, showing a concentration dependent inhibition zone for both free and microencapsulated EO's, which is in agreement with the hypothesis that Gram-positive bacteria are more sensitive to plants phytochemicals [31–33]. In addition, this microorganism has shown sensitivity to thymol, carvacrol, eugenol, *α*-pinene, *β*-pinene, linalool, terpinen-4-ol, and terpineol [5], which are components of MXO and EUO essential oils (Table 1). Because of the high cost of individual compounds, the use of complex EO's remains a good choice to be used as part of the antimicrobial barriers used in food safety.

Size of inhibition zone can be affected by chemical composition of the EO's [3], rate of diffusion into the agar, and chemicals volatility [12], which may have affected the applied dose to the PVDF membranes [34] (Table 5).

*L. plantarum* was not sensitive to free or microencapsulated EUO essential oil, probably associated with its low thymol peak area composition (Table 1), in contrast to free or microencapsulated MXO essential oil (Table 5) which shows thymol as its major component. Thymol has been reported as highly effective against this microorganism [5].

*B. thermosphacta *was more sensitive to microencapsulated than free MXO essential oil (Table 5), probably because of variations in the relative proportion of phenolic compounds due to the microencapsulation process (Table 2). Free and microencapsulated EUO EO's were effective only at high concentration (25% w/v) (Table 5). Several compounds present in MXO and EUO essential oils have inhibited* B. thermosphacta *growth; they include eugenol, geranyl acetate, thymol, linalool, *β*-pinene, carvacrol, terpinen-4-ol, and terpineol (Table 1), thymol and carvacrol being the most effective compounds [5].

*Salmonella *was sensitive to both concentrations of MXO and EUO essential oils, while, for microencapsulated samples, only MXO essential oil was active at 25% concentration, which may be attributed to correspondingly higher amounts of active compounds. Free and microencapsulated MXO essential oils at both concentrations tested were effective to inhibit* Pseudomona fragi*, whereas free and microencapsulated EUO essential oil were only effective at 25% concentration, despite that this genus has consistently shown high resistance to phenolic antimicrobial compounds [35]. In addition, coriander essential oil fractions containing mainly *α*-pinene, camphene, and linalool were reported to show inhibitory effect on* Pseudomona fragi* [32]. However, these compounds are in higher concentration in EUO than in MXO essential oils, but interactions among components may have affected EUO essential oil effectiveness. According to Lambert et al. [9], the additive effect of thymol and carvacrol from EUO accounted for 96% inhibition against* Pseudomona aeruginosa*. Microencapsulated MXO essential oil was more effective against* Pseudomona fragi* and* Salmonella* sp. than free and microencapsulated EUO essential oils, which is probably associated with higher thymol and carvacrol composition (Table 2). In addition, Gram-negative bacteria such as* S. typhimurium* are more sensitive to thymol than to carvacrol [36]. Other compounds such as eugenol, geranyl acetate, *β*-pinene, terpinene, terpinen-4-ol, terpineol, and *α*-terpinolene (Table 1) have shown antibacterial activity against* Pseudomonas* and* Salmonella* genera [5, 32].

Due to the large number of different groups of compounds present in EO's, their antibacterial activity is not attributable to one specific mechanism. Thymol, carvacrol, and eugenol are membrane permeabilizers causing leakage of various substances, such as ions, ATP, nucleic acids, and amino acids; *α*-pinene, terpinene, and *β*-pinene disrupt the structure of cytoplasm membrane, inhibiting electron transport, and linalool, terpinen-4-ol, and terpineol potentially act as either protein denaturing agents, solvents, or dehydrating agents [5, 9]. More studies are needed to determine the mechanism of action of individual compounds of EO's against specific microorganisms and to carry out antimicrobial tests on wider microbial spectra.

### 3.7. Essential Oils Stability

After three months of refrigerated storage, the antibacterial effect of MXO and EUO free essential oils against* Micrococcus luteus *was 59.3% and 33.3% of the initial value, respectively (Table 6). However, microencapsulated essential oils did not experience changes in their antibacterial activity. Microencapsulation protected bioactive components of EO's, retaining their antimicrobial activity.

## 4. Conclusion

Detailed compositional analysis was achieved by GC/MS on diluted EO's using two different solvents, and it was demonstrated that the solvent used affected the qualitative and quantitative composition. Composition of microencapsulated EO's was evaluated by HS-SPME, which showed that MXO contained high proportion of thymol and carvacrol. A combination of these two compounds showed additive effect against* M. luteus.* EO's antimicrobial activity depended on type and microorganisms tested. MXO essential oil was an effective antimicrobial inhibitor. Stability of essential oils was enhanced by a microencapsulation process, where most antimicrobial activity was retained, leading to water soluble microcapsules with reduced aroma. These characteristics may be useful when incorporating the EO's in food systems.

## Figures and Tables

**Table 1:** Composition of *Origanum  vulgare* and *Lippia  graveolens* essential oils diluted with methanol and pentane^a^.

Number	Compound	Composition (% peak area)				KI^b^	KI^c^	Identification	Reference
		*Lippia* Methanol	*Lippia* Pentane	*Origanum* Methanol	*Origanum* Pentane				
1	Dimethyl sulfide	0.00		0.01		515	505	KI, MS	[37]
2	2-Methyl-propanal			0.01		547	552	KI, MS	[38]
3	Cyclopentane		0.18			551	560	KI, MS	[38]
4	Trichloromethane			0.001		614	616	KI, MS	[38]
5	3-Methyl-butanal		0.01	0.01	0.01	644	649	KI, MS	[38]
6	2-Methyl-butanal		0.01	0.01		654	658	KI, MS	[38]
7	2-Ethyl-furan	0.001		0.01	0.01	696	691	KI, MS	[38]
8	2-Methyl-propanoic acid ethyl ester	0.01	0.01			757	755	KI, MS	[38]
9	Acetic acid 2-methylpropyl ester	0.01	0.02			774	779	KI, MS	[38]
10	2-Methyl-butanoic acid methyl ester	0.01		0.01		777	780	KI, MS	[38]
11	3-Methyl-2-butenal	0.01	0.01			785	783.3	KI, MS	[38]
12	2-Methyl-butanoic acid ethyl ester	0.03	0.03			852	848	KI, MS	[37]
13	2-Hexenal	0.05		0.03	0.03	857	854	KI, MS	[38]
14	3-Methyl-1-butanol acetate	0.03	0.03			878	876	KI, MS	[38]
15	3-Methyl-3-buten-1-ol acetate	0.02				885	878	KI, MS	[38]
16	*α*-Pinene	4.85	2.30	15.56	10.38	925	926	KI, MS	[7]
17	Dehydrosabinene			0.02		948	957	KI, MS	[38]
18	Camphene	0.04	0.04	0.10	0.08	955	954	KI, MS	[38]
19	Benzaldehyde	0.01	0.01			968	970	KI, MS	[38]
20	*β*-Thujene	2.08	2.32	2.90	2.59	977	977	KI, MS	[38]
21	*β*-Pinene	0.11	0.26	0.39	0.33	982	981	KI, MS	[38]
22	3-Octanone			0.09	0.07	987	985	KI, MS	[38]
23	*β*-Myrcene		2.32			991	991	KI, MS	[38]
24	2-Methyl-butanoic acid-2-methylpropyl ester	0.01				1003	1004	KI, MS	[38]
25	*α*-Phellandrene	0.25	0.02	0.39	0.34	1010	1007	KI, MS	[38]
26	3-Carene	0.23	0.001	0.29	0.001	1012	1011	KI, MS	[38]
27	*α*-Terpinene	2.02	2.32	6.95	5.88	1022	1024	KI, MS	[38]
28	*o*-Cymene			5.11		1031	1027	KI, MS	[38]
29	*p*-Cymene				4.42	1031	1029	KI, MS	[38]
30	D-Limonene	0.32	0.37	1.05	0.89	1035	1031	KI, MS	[38]
31	*trans*-*β*-Ocimene			5.10	3.96	1039	1041	KI, MS	[38]
32	Eucalyptol	1.02	0.97			1040	1036	KI, MS	[38]
33	*cis*-*β*-Ocimene		0.13		0.57	1042	1040	KI, MS	[38]
34	Butyl 2-methylbutanoate		0.01			1043	1043	KI, MS	[39]
35	*β*-Ocimene	0.13		0.69	0.57	1049	1050	KI, MS	[38]
36	*γ*-Terpinene	9.59	10.31	0.21	15.06	1068	1063	KI, MS	[38]
37	Terpinolene	0.17	0.18	2.26	1.84	1090	1089	KI, MS	[38]
38	*p*-Cymenene	0.06	0.05	0.04	0.03	1095	1088	KI, MS	[38]
39	*β*-Linalool	0.70	0.61	1.85	1.51	1102	1100	KI, MS	[38]
40	1-Octen-3-yl-acetate			0.23		1107	1110	KI, MS	[38]
41	*cis*-Sabinene-hydrate		0.08			1108	1105	KI, MS	[11]
42	neo-allo-Ocimene	0.01	0.01		0.74	1130	1129	KI, MS	[39]
43	*trans*-allo-Ocimene			0.14		1131	1129	KI, MS	[39]
44	*trans*-Menth-2-en-1-ol	0.04		1.02	0.82	1133	1140	KI, MS	[38]
45	*s*-Ipsdienol	0.30	0.26			1156	1147	KI, MS	[39]
46	*m*-Xylidine		0.01			1167	1168	KI, MS	[39]
47	Pinocarvone			0.03	0.02	1173	1168	KI, MS	[39]
48	3-Thujene-2-one	0.10	0.42			1178	1168	KI, MS	[38]
49	Terpinen-4-ol	0.59	0.56	14.77	12.40	1190	1187	KI, MS	[38]
50	*α*-Terpineol	0.19	0.17	2.32	1.89	1206	1203	KI, MS	[38]
51	*trans*-Piperitol			0.17	0.30	1219	1212	KI, MS	[38]
52	Thymol methyl ether	1.32	1.16	2.54	2.06	1235	1235	KI, MS	[38]
53	Isothymol methyl ether	0.10	0.08	3.20		1245	1244	KI, MS	[38]
54	Linalyl acetate			0.80	0.56	1252	1251	KI, MS	[38]
55	Thymol	66.30	49.90	9.30	11.50	1288	1290	KI, MS	[38]
56	Carvacrol	0.10	0.10	8.40	8.40	1300	1299	KI, MS	[38]
57	Bornyl acetate			0.24		1303	1297	KI, MS	[38]
58	Thymol acetate	0.07	0.05			1353	1352	KI, MS	[38]
59	Eugenol		0.07	0.03		1362	1362	KI, MS	[38]
60	Geranyl acetate			0.13	0.13	1379	1383	KI, MS	[39]
61	Ylangene		0.01			1384	1375	KI, MS	[3]
62	Copaene	0.03	0.02			1390	1382	KI, MS	[3]
63	*β*-Elemene	0.06				1401	1405	KI, MS	[38]
64	Isocaryophyllene				0.01	1422	1413	KI, MS	[39]
65	*β*-*cis*-Caryophyllene	0.01				1422	1428	KI, MS	[38]
66	Caryophyllene	1.87	1.64	2.15	1.74	1441	1444	KI, MS	[38]
67	*trans*-*α*-Bergamotene	0.27	0.23			1445	1436	KI, MS	[38]
68	Farnesyl acetone B	0.04	0.03			1453	1453	KI, MS	[38]
69	*cis*-*β*-Farnesene	0.05	0.04			1457	1457	KI, MS	[38]
70	*α*-Himachalene			0.06		1459	1460	KI, MS	[3]
71	Humulene	1.22	1.04	0.30	0.24	1477	1477	KI, MS	[38]
72	Alloaromadendrene			0.05		1482	1478	KI, MS	[39]
73	*α*-Muurolene		0.06	0.12	0.08	1492	1493	KI, MS	[38]
74	Germacrene D	0.04			0.71	1502	1503	KI, MS	[38]
75	*δ*-Guaiene			0.14		1510	1506	KI, MS	[38]
76	*β*-Selinene	0.04	0.03			1511	1509	KI, MS	[38]
77	7-epi-*α*-Cadinene	0.02	0.02			1514	1522	KI, MS	[39]
78	*β*-Bisabolene	0.76	0.64			1519	1509	KI, MS	[38]
79	*δ*-Cadinene	0.07	0.04	0.10	0.08	1533	1525	KI, MS	[38]
80	Calamenene	0.03	0.02			1539	1531	KI, MS	[3]
81	Nerolidol	0.12	0.10			1567	1566	KI, MS	[38]
82	Spathulenol			0.35	0.26	1598	1596	KI, MS	[24]
83	Caryophyllene oxide	0.67	0.41	0.27	0.20	1606	1613	KI, MS	[38]
84	Viridiflorol			0.04	0.03	1618	1620	KI, MS	[38]
85	*γ*-Eudesmol	0.01	0.01			1653	1646	KI, MS	[38]
86	*α*-Cadinol	0.03	0.02	0.03	0.02	1676	1669	KI, MS	[38]
87	6,10,14-Trimethyl-2-pentadecanone		0.001			1851	1856	KI, MS	[38]
Grouped components (%):									
Monoterpene hydrocarbons		23	25	31	39				
Oxygenated monoterpenes		25	26	29	27				
Sesquiterpene hydrocarbons		23	21	14	14				
Oxygenated sesquiterpenes		7	7	8	10				
Others		22	21	18	10				

^a^Compositions are mean of three replicates with standard error within 5% of the mean.

^b^Calculated *Kovats* retention indices.

^c^ *Kovats* retention indices reported in literature.

**Table 2:** Headspace volatile compounds identified from microcapsules of *Lippia  graveolens* H.B.K. and *Origanum  vulgare* L. essential oils by headspace extraction.

Number	Compound	Composition (area units^a^ × 10^6^)		KI^b^	KI^c^	Identification method	Reference
		*Lippia graveolens *	*Origanum vulgare *				
1	2-Ethyl-furan	0.70	1.55	696	691	KI, MS	[38]
2	2-Methyl-butanoic acid ethyl ester	1.12		852	848	KI, MS	[37]
3	*α*-Pinene	42.64	552.28	925	926	KI, MS	[7]
4	Camphene	5.83	17.58	955	954	KI, MS	[38]
5	Benzaldehyde	1.79		968	970	KI, MS	[38]
6	*β*-Pinene	455.61		982	981	KI, MS	[38]
7	*α*-Phellandrene	26.34	254.41	1010	1007	KI, MS	[38]
8	3-Carene	3.21	620.39	1012	1011	KI, MS	[38]
9	*α*-Terpinene	379.89		1022	1024	KI, MS	[38]
10	*p*-Cymene		1297.17	1031	1029	KI, MS	[38]
11	D-Limonene	31.88		1035	1031	KI, MS	[38]
12	*β*-Ocimene	10.11	133.89	1049	1050	KI, MS	[38]
13	*γ*-Terpinene	1901.53	3937.65	1068	1063	KI, MS	[38]
14	Terpinolene	3.40	2343.48	1090	1089	KI, MS	[38]
15	neo-allo-Ocimene		9.86	1130	1129	KI, MS	[39]
16	*α*-Terpineol		101.54	1206	1203	KI, MS	[38]
17	Thymol methyl ether	163.36	153.34	1235	1235	KI, MS	[38]
18	Isothymol methyl ether		1467.36	1245	1244	KI, MS	[38]
19	Thymol	1097.05	171.95	1288	1290	KI, MS	[38]
20	Carvacrol	2297.22	255.86	1300	1299	KI, MS	[38]
21	Eugenol	1.78		1362	1362	KI, MS	[38]
22	Ylangene	0.62		1384	1375	KI, MS	[3]
23	Copaene	1.76		1390	1382	KI, MS	[3]
24	Caryophyllene	121.66	301.65	1441	1444	KI, MS	[38]
25	*trans*-*α*-Bergamotene	13.13		1445	1436	KI, MS	[38]
26	Humulene	58.29	31.96	1477	1477	KI, MS	[38]
27	Alloaromadendrene		14.72	1482	1478	KI, MS	[39]
28	Germacrene D		76.27	1502	1503	KI, MS	[38]
29	*β*-Bisabolene	20.98		1519	1509	KI, MS	[38]
30	*δ*-Cadinene	1.85	10.04	1533	1525	KI, MS	[38]

^a^Area unit is equivalent to chromatographic peak area produced for each compound.

^b^Calculated *Kovats* retention indices.

^c^ *Kovats* retention indices reported in literature.

**Table 3:** Free and microencapsulated thymol and carvacrol antimicrobial activity against *Micrococcus  luteus* determined by the broth dilution method. After 2 hrs incubation at 30°C.

Antimicrobial compound	Concentration (*µ*g/mL)	Form	
		Free (log CFU/mL)	Microencapsulated (log CFU/mL)
Thymol	0	5.51^Aa^ ± 0.12	5.52^Aa^ ± 0.03
	50	4.17^Bb^ ± 0.11	5.44^Aa^ ± 0.11
	150	3.67^Ca^ ± 0.18	1.70^Bb^ ± 0
	250	3.58^Ca^ ± 0.12	1.70^Bb^ ± 0

Carvacrol	0	5.51^Aa^ ± 0.12	5.52^Aa^ ± 0.03
	80	5.49^Aa^ ± 0.08	4.46^Bb^ ± 0.15
	100	5.33^Aa^ ± 0.30	1.70^Cb^ ± 0
	300	5.22^Aa^ ± 0.24	1.70^Cb^ ± 0
	500	3.34^Ba^ ± 0.24	1.70^Cb^ ± 0

Different capital superscript letters indicate significant difference (*P* < 0.05) within a column, whereas different lowercase superscript letters indicate significant difference (*P* < 0.05) within rows.

**Table 4:** Combined effect of thymol and carvacrol to achieve inhibition of *Micrococcus  luteus*.

Thymol MIC (*µ*g/mL)	Carvacrol MIC (*µ*g/mL)	Combination (*µ*g/mL)		Thymol FIC	Carvacrol FIC	FIC_index_
		Thymol	Carvacrol			
1250	1000	750	500	0.6	0.5	1.1
		750	300	0.6	0.3	0.9
		1000	300	0.8	0.3	1.1

**Table 5:** Inhibition zone diameters obtained with *Lippia  graveolens* H.B.K. and *Origanum  vulgare* L. essential oils at 15% and 25% (w/v) concentration against test organisms.

Microorganism	Concentration of essential oil (%)	MXO essential oil (mm)	Microencapsulated MXO essential oil (mm)	EUO essential oil (mm)	Microencapsulated EUO essential oil (mm)
*Salmonella *sp.	15	26.35^aB^ ± 0.84	0^cB^	24.96^bB^ ± 0.06	0^cA^
	25	29.23^aA^ ± 0.18	24.85^cA^ ± 0.26	27.88^bA^ ± 0.16	0^dA^
*Brochothrix thermosphacta *	15	0^bA^	24.43^aA^ ± 0.21	0^bB^	0^bB^
	25	0^bA^	24.82^aA^ ± 0.14	24.96^aA^ ± 0.02	24.58^aA^ ± 0.33
*Pseudomonas fragi *	15	24.95^aA^ ± 0.01	24.82^bA^ ± 0.10	0^cB^	0^cB^
	25	24.92^aB^ ± 0.01	24.91^aA^ ± 0.35	24.93^aA^ ± 0.03	24.67^aA^ ± 0.25
*Lactobacillus plantarum *	15	24.87^aB^ ± 0.02	24.93^aA^ ± 0.21	0^bA^	0^bA^
	25	24.94^aA^ ± 0.01	25.02^aA^ ± 0.23	0^bA^	0^bA^
*Micrococcus luteus *	15	67.69^aB^ ± 0.19	32.27^bB^ ± 0.48	27.12^cB^ ± 1.28	24.93^dB^ ± 0.18
	25	83.08^aA^ ± 0.09	43.58^cA^ ± 0.08	75.19^bA^ ± 0.10	28.33^dA^ ± 0.81

a–d: for each test microorganism, means within a row not having a common superscript letter are different (*P* < 0.05).

A and B: for each test microorganism, means within a column (between concentrations) not having a common superscript letter are different (*P* < 0.05).

**Table 6:** Stability of free and microencapsulated *Lippia  graveolens* H.B.K. and *Origanum  vulgare* L. essential oils at 25% at 4°C. Antimicrobial activity is shown as inhibition zone (mm) using *Micrococcus  luteus*.

Refrigerated storage (months)	MXO essential oil (mm)	Microencapsulated MXO essential oil (mm)	EUO essential oil (mm)	Microencapsulated EUO essential oil (mm)
0	83.08^a^ ± 0.09	43.58^a^ ± 0.08	75.19^a^ ± 0.1	28.33^a^ ± 0.81
3	49.27^b^ ± 0.99	42.74^a^ ± 1.30	25^b^ ± 0.01	28.17^a^ ± 1.01

Means within a column not showing a common superscript letter are different (*P* < 0.05).
